# A decisive year to International Brazilian Journal of Urology

**DOI:** 10.1590/S1677-5538.IBJU.2021.01.01

**Published:** 2020-11-18

**Affiliations:** 1 Universidade do Estado de Rio de Janeiro Unidade de Pesquisa Urogenital Rio de JaneiroRJ Brasil Unidade de Pesquisa Urogenital - Universidade do Estado de Rio de Janeiro - Uerj, Rio de Janeiro, RJ, Brasil; 2 Hospital Federal da Lagoa Serviço de Urologia Rio de JaneiroRJ Brasil Serviço de Urologia, Hospital Federal da Lagoa, Rio de Janeiro, RJ, Brasil

## COMMENT

The year of 2020, my first year as Editor-in-Chief of International Brazilian Journal of Urology, was very difficult due to the COVID-19 outbreak, but the quarantine period led to an increase in the number of submissions for our journal and we had a significant increase in our impact factor in June 2020. During this period, we ended up with the retention of articles, increased the rigor of selection and decreased the average rating of articles from 47 days to 22 days. In 2021 we will continue to work hard to increase the impact of our Journal. The January-February number of Int Braz J Urol, the seventh under my supervision, presents original contributions with a lot of interesting papers in different fields: Prostate Cancer, Male Infertility, Female Incontinence, Renal Cell Carcinoma, Urinary Stones, Testicular migration, Laparoscopy, BPH, Partial Nephrectomy, Nocturnal Enuresis and Bladder Cancer. The papers came from many different countries such as Brazil, USA, Turkey, China, Republic of Korea, Chile, UK, India, Spain and Iran, and as usual the editor´s comment highlights some of them.

In the present issue we present three important papers about Male Infertility. Dr. Bin and colleagues from China performed in page 8 (1) a nice systematic review about the association between body mass index and varicocele and concluded that body mass index is negatively associated with the presence of varicocele. Dr. Cicek and colleagues from Turkey present in page 112 (2) an important study about the association between seminal oxidation reduction potential (ORP) and conventional sperm parameters and shows that the presence of oligozoospermia, reduced progressive motilty or low total motility sperm count in sperm analysis should raise the suspicion of oxidative stress and warrants seminal reactive oxygen species testing and Dr. Groner et al. in page 185 (3) performed on Expert Opinon section an interesting mini-review about the effects of Covid-19 on male reproductive system and suggested that the involvement of the male reproductive system which could be a new route of the disease transmission. The virus has already been found in the semen of infected patients, but it remains to be assessed what impacts it has on male reproductive health. The editor in chief would like to highlight the following works too:

Dr. Ouyang and collegues from China presented in page 23 (4) a nice systematic review about medical expulsive therapy and ESWL and shows that Adjunctive medical expulsive therapy with tamsulosin is effective in patients with specific stone size or location that received repeated ESWL. However, no well-designed randomized controlled trial that used computed tomography for the detection and assessment of residual stone fragments was found.

Dr. Deng and collegues from China (5) shows an amazing meta-analysis on page 46 about surgical technique in pathological T3a renal cell carcinoma (RCC) and shows that partial nephrectomy may be more suitable for treating pT3a RCC than radical nephrectomy because it provides a similar survival time (OS or RFS) and superior renal function.

Dr. Gökce and collegues (6) from Turkey performed on page 64 a interesting study comparing the retrograde ureterorenoscopy (URS) and percutaneous anterograde ureteroscopy for removal of impacted upper ureteral stones >10mm in the elderly population and concluded that the anterograde URS in supine position provided better success rates and similar complication rates compared to retrograde URS.

Dr. Ghanavati and collegues (7) from Iran performed a randomized controlled clinical trial on page 73 an interesting study about primary nocturnal enuresis (PMNE) and shows that the combination of desmopressin and an anticholinergic agent is highly effective in treatment of children with PMNE. Although desmopressin has long been a first-line treatment for PMNE, desmopressin monotherapy often fails to achieve a successful response in patients with PMNE.

Dr. Mercimek and collegues (8) from Turkey developed on page 103 a study about the off-clamp laparoscopic partial nephrectomy (Off-C LPN) access techniques and concluded that transperitoneal and retroperitoneal access were found to have similar outcomes in terms of preservation of renal function at the end of the first year postoperatively. Off-C LPN may be considered as a safe and effective treatment option in patients having non-complex renal tumors.

Dr. Ribeiro and collegues (9) from Brazil analyzed on page 120 a study about the pelvic floor muscles after prostate radiation therapy and the morpho-functional assessment by magnetic resonance imaging, surface electromyography and digital anal palpation and concluded that no changes were observed in the morpho-functional parameters evaluated by MRI, except the measurement of the membranous urethra length when comparing Pre-RT Group and Acute and Late Groups.

Dr. Otaola-Arca and collegues (10) from Spain and Chile studied on page 131 the clinical efficacy and safety profile between monopolar transurethral resection of the prostate (M-TURP) and bipolar plasmakinetic resection of the prostate (PK-TURP) for benign prostatic hyperplasia (BPH) and concluded that there is not significant variation in effectiveness and safety between M-TURP and PK-TURP for the treatment of BPH.

Dr. Netto and collegues (11) from Brazil performed on page 169 an interesting study about the personal and familial factors associated with toilet training (TT) and concluded that children completed TT at a mean of 2 years and 7 months of age. The age of completing TT was not related to LUTS and/or constipation. Premature children and those whose mothers work outside the home finish TT later.

The Editor-in-chief expects everyone to enjoy reading and for sure better times will come soon.
